# Commentary: Lethal Pneumonia Cases in Mojiang Miners (2012) and the Mineshaft Could Provide Important Clues to the Origin of SARS-CoV-2

**DOI:** 10.3389/fpubh.2021.702199

**Published:** 2021-07-13

**Authors:** Alex C. Speciale

**Affiliations:** Independent Researcher, Los Angeles, CA, United States

**Keywords:** SARS-CoV-2, COVID-19, mineshaft, RaTG13, outbreak, Mojiang, origin, spillover

## Introduction

It has been more than 1 year since the Covid-19 pandemic began in 2020 (1). The evolutionary origins of the SARS-CoV-2 virus can be partially traced to a lineage in bats; the closest known relative to SARS-CoV-2 (RaTG13) was found in a bat fecal sample taken from an abandoned mineshaft in Mojiang, China (2, 3). SARS-CoV-2 is proposed to have evolved via an intermediate host between bats and humans, or through natural selection in humans through direct contact with bats (2). Following a WHO-China investigation into the origins of the pandemic, a letter published in Science has pointed out analytical drawbacks of the report, that further investigatory efforts are needed to pinpoint the origin of SARS-CoV-2, and that both natural and laboratory spillover hypotheses must be taken seriously (4). Confirmation on the evolutionary origins of SARS-CoV-2 is critical for preventing future zoonotic outbreaks and pandemics.

In October 2020, an article by Rahalkar and Bahulikar was published in Frontiers in Public Health (5) that raises questions about a 2012 lethal pneumonia outbreak and the origin of SARS-CoV-2. Almost 1 month after the publication of the paper in Frontiers in Public Health, more new details were published in Nature (3) by researchers at the Wuhan Institute of Virology (WIV) that seem to contradict the findings presented by Rahalkar and Bahulikar. The emergence of this information and of the possible contradictions that have been made since, present critical, global public health implications and outline the need for clarification. This situation gives insight into obligatory, ethical steps that are to be considered for discovering the origin of the Covid-19 pandemic, and for preventing future pandemics.

## Lethal Pneumonia Outbreak in Mojiang Miners

Rahalkar and Bahulikar bring to light a 2012 lethal pneumonia outbreak (5) that occurred among 6 miners in Mojiang, China, who were working in the same mine that RaTG13 was collected from following the outbreak (3). Bat fecal samples were taken from the mine by a team of researchers from WIV primarily because bat virus transmission was suspected as the cause of outbreak (3). Indeed, the miners were exposed to bats of the genus *Rhinolophus* that are known to harbor SARS-like coronaviruses (5). While molecular studies on RatG13 suggest that transmission to humans and subsequent evolution of SARS-CoV-2 is unlikely to have occurred under normal ecological conditions (2), there are a number of possibilities as to how the Mojiang mining incident could be related to the current Covid-19 pandemic. For example, it is possible that a closer, less virulent relative to SARS-CoV-2 infected the miners and was transmitted to the local population in Yunnan Province where the miners sought medical care, eventually evolving into SARS-CoV-2 via natural selection in the regional human population. Alternatively, Dr. Jonathan Latham and Dr. Allison Wilson propose that the pre-existing conditions of the miners' lungs may have presented an unordinary opportunity for viral entry into lung tissue, which could have resulted in rapid evolution. Latham and Wilson call this the Mojiang Miners Passaging (MMP) hypothesis^1^. The bats in the mine were found to have co-infections of coronaviruses which further adds to the plausibility of the MMP hypothesis, as co-infections can make viruses more transmissible and pathogenic (6).

## Miners' Sample Results

Samples taken from the miners were sent to Chen Du army reserved Center for Disease Prevention and Control (7) and WIV (5) for testing in 2012. Latham and Wilson propose that it is possible SARS-CoV-2 was present in one or more of these samples and accidentally escaped from WIV.^1^ In this scenario, an unknown intermediate SARS-like CoV more closely related to SARS-CoV-2 than RaTG13 might have infected the miners, evolved into SARS-CoV-2, and leaked from the samples. Laboratory and natural spillover hypotheses can be tested by sequencing and retesting the mentioned samples for SARS-CoV-2 and other SARS-like CoV positivity. To our knowledge, some testing has already been done. However, the results among the sources raise some concerns.

Approximately 1 month after the Rahalkar and Bahulikar article was published in Frontiers in Public Health (5), an addendum to the 2020 paper by researchers at the WIV that first reported sequencing of the SARS-CoV-2 genome (8), states that WIV received 13 samples from 4 of the miners in 2012 (3). They write that in 2012 they used PCR methods that target RNA-dependent RNA polymerase (RdRp) of SARSr-CoV Rp3 to test the samples and all four individuals showed negative results (3). The addendum by WIV researchers further states that they retested the samples in 2020 with their validated enzyme-linked immunosorbent assay (ELISA) against the SARS-CoV-2 nucleocapsid protein and all tests were negative (3). However, a Chinese PhD thesis published in 2017 by Canping Huang is mentioned in the Rahalkar and Bahulikar article, and states that antibody testing conducted on the miners' samples revealed that four of the miners tested positive for “SARS virus IgG antibodies” (5). Due to observed robust cross- reactive neutralization of SARS-CoV-2 by serum antibodies from recovered SARS patients (9), it is particularly curious why the SARS-CoV-2 ELISA assay used by WIV did not show positive results with the presence of SARS virus IgG antibodies, as indicated by the PhD thesis. At the very least, those samples should have been analyzed for other SARS-like CoV viruses. Furthermore, Rahalkar and Bahulikar discuss a Master's thesis that was also published in Chinese, and that focused on analyzing the illnesses of the miners in great detail. The Master's thesis states that one of the patients tested positive for serum IgM by WIV, which can indicate viral infection, and concludes that the illness among the miners was likely caused by a SARS-like CoV virus of bat origin (7). The Master's thesis has since been translated into English^2^. Oddly, the addendum by WIV researchers (3) and the WHO-China report^3^ made no direct comments or acknowledgments on the important findings of the PhD and Master's theses, nor on other critical questions presented by Rahalkar and Bahulikar. As pointed out by Rahalkar and Bahulikar, the miners' symptoms were strikingly similar to Covid-19 symptoms (5).

## Discussion

The information outlined in the Rahalkar and Bahulikar article that revealed the Mojiang miners tested positive for SARS IgG antibodies, the sample analyses information given by WIV, and the aforementioned hypotheses, present a situation for obligatory scientific questions to be asked and ethical considerations to be taken into account. A number of pertinent questions asked by Rahalkar and Bahulikar still remain unanswered. For example, what is the availability of the miners' samples to be studied by other researchers, and is there any DNA/RNA available from the samples? Why was the outbreak not reported to WHO, nor mentioned by PREDICT? A new question to be addressed is why did the PhD thesis report SARS virus IgG antibody positivity for the miners' samples, while the WIV reported negative tests? Another peculiarity is that the WIV made numerous trips to a distant mineshaft from 2012 to 2015 to collect and analyze viral samples (3), yet they report rather limited testing on the miners' samples, that were thought to contain highly virulent pathogens, and that have been in the lab's possession since 2012. Surely these samples would have been of interest to a lab specializing in coronavirus zoonosis, yet no sequencing has been reported.

From a responsible public health point of view, it is critical that the mentioned SARS virus IgG positive samples and other collected samples mentioned in the Master's thesis be located, verified, sequenced, and re-examined by stakeholders who have no conflicts of interest. Pertaining to the relevant laboratories, determination on the tests and experimental studies conducted on the miners' samples, as well as on naturally collected and artificially passaged coronavirus samples should be investigated. As noted by Bloom and colleagues, hypotheses involving natural and lab spillover should be equally considered, and “investigators should document the veracity and provenance of data from which analyses are conducted and conclusions drawn, so that analyses are reproducible by independent experts”(4). Consistent with the plausibility of a lab spillover event, is the recent US government fact sheet that reveals there is “reason to believe that several researchers inside the WIV became sick in autumn 2019, before the first identified case of the outbreak, with symptoms consistent with both COVID-19 and common seasonal illnesses”^4^. However, evidence that validates that claim has not been provided to the public. Additionally, continued sampling of the bats in the Mojiang mineshaft may reveal SARS-like coronaviruses that are closely related to SARS-CoV-2. There are numerous hypotheses to be explored regarding the origin of^4^ SARS-CoV-2, and given the global health implications, it is critical that scientific inquiry into the origin of SARS-CoV-2 not be stifled by sociocultural or ideological factors. Lastly, the WHO-China report states that the team supports continued scientific and collaborative efforts to be employed for discovering the origin of the pandemic, and that to increase knowledge regarding a lab origin, follow-up of new evidence of a possible lab leak would be needed^3^; new evidence for follow-up is provided herein.

## Author Contributions

AS: conception of ideas, design, writing, editing, and obtainment of scientific sources.

## Conflict of Interest

The author declares that the research was conducted in the absence of any commercial or financial relationships that could be construed as a potential conflict of interest.

## Footnotes

^1^Available online at: https://www.independentsciencenews.org/commentaries/a-proposed-origin-for-sars-cov-2-and-the-covid-19-pandemic/

^2^Available online at: https://www.documentcloud.org/documents/6981198-Analysis-of-Six-Patients-With-Unknown-Viruses.html

^3^Available online at: https://www.who.int/publications/i/item/who-convened-global-study-of-origins-of-sars-cov-2-china-part

^4^Available online at: https://2017-2021.state.gov/fact-sheet-activity-at-the-wuhan-institute-of-virology/index.html
